# The Emerging Role of Major Regulatory RNAs in Cancer Control

**DOI:** 10.3389/fonc.2019.00920

**Published:** 2019-09-24

**Authors:** Xiaofeng Dai, Aman Chandra Kaushik, Jianying Zhang

**Affiliations:** ^1^Wuxi School of Medicine, Jiangnan University, Wuxi, China; ^2^School of Life Sciences and Biotechnology, Shanghai Jiao Tong University, Shanghai, China; ^3^Henan Key Laboratory of Tumor Epidemiology, Henan Institute of Medical and Pharmaceutical Sciences, Zhengzhou University, Zhengzhou, China

**Keywords:** long non-coding RNA, microRNA, biomarker, therapeutics, cancer

## Abstract

Alterations and personal variations of RNA interactions have been mechanistically coupled with disease etiology and phenotypical variations. RNA biomarkers, RNA mimics, and RNA antagonists have been developed for diagnostic, prognostic, and therapeutic uses. Long non-coding RNAs (lncRNAs) and microRNAs (miRNAs) are two major types of RNA molecules with regulatory roles, deregulation of which has been implicated in the initiation and progression of many human malignancies. Accumulating evidence indicated the clinical roles of regulatory RNAs in cancer control, stimulating a surge in exploring the functionalities of regulatory RNAs for improved understanding on disease pathogenesis and management. In this review, we highlight the critical roles of lncRNAs and miRNAs played in tumorigenesis, scrutinize their potential functionalities as diagnostic/prognostic biomarkers and/or therapeutic targets in clinics, outline opportunities that ncRNAs may bring to complement current clinical practice for improved cancer management and identify challenges faced by translating frontier knowledge on non-coding RNAs (ncRNAs) to bedside clinics as well as possible solutions.

## Introduction

Non-coding RNAs (ncRNAs) are RNA molecules with no protein translation potential. The conception that ncRNAs are “junk RNAs” of the transcriptome has been subverted by the fact that an essential part of the genome is transcribed into RNAs without protein products, with the rapid development of high-throughput technologies (1, 2). NcRNAs can be classified as housekeeping ncRNAs for the maintenance of normal cell functionalities, and regulatory ncRNAs. House keeping ncRNAs include, e.g., snoRNAs (small nucleolar RNAs), snRNAs (small nuclear RNAs), gRNA (guide RNAs), RNaseP RNAs that take part in processing transcriptional products, and tRNAs (transfer RNAs), rRNAs (ribosome RNAs), and tmRNAs (transfer messenger RNAs) that are involved in protein translation, as well as telomere related RNAs and SRP RNAs (signal recognition particle RNAs). A diverse reservoir of ncRNAs with regulatory roles on gene expression or cellular events has been revealed including, e.g., long ncRNAs (lncRNAs), microRNAs (miRNAs), piwi interaction RNAs (piRNAs), and circular RNAs (circRNAs). This review focuses on regulatory ncRNAs, whose indispensible roles on maintaining the special-temporal architecture of transcriptional and translational programs under healthy and malignant states have been gaining incremental attentions.

NcRNAs modulate gene expression through various mechanistic programs. NcRNA-mediated gene silencing constitutes one important type of epigenetic alterations and has been implicated in several human carcinogenesis (3). An increasing number of studies have uncovered associations between ncRNAs and cancer predisposition or status during the past decade (4), opening a new paradigm for cancer control taking advantages of regulatory RNAs. Among the ever-increasing types of ncRNAs being deciphered, lncRNAs and miRNAs are the most intensively studied (5). In particular, lncRNAs, frequently found deregulated in various types of cancers, represent a novel goldmine for biomarker discovery as well as therapeutic applications (6–9); miRNAs have been subsequently identified dysregulated in almost all types of cancers and proposed for diagnosis and therapeutics ever since the discovery of the loss-of-function phenotypes conveyed by the miRNA let-7 (10). For instance, lncRNA *LINC00261* was reported to suppress cell proliferation and invasion in human choriocarcinoma (11), and miR-21 was implicated as an oncogenic factor regulating cancer cell proliferation, migration and apoptosis in many diseases including cancers (12, 13).

**Figure 1:**
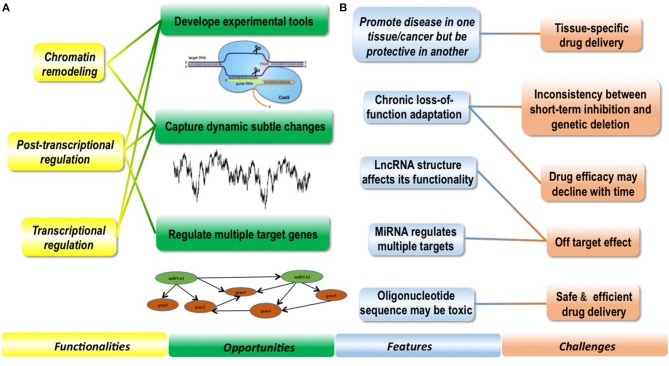
Conceptual scheme illustrating the features and functionalities of regulatory ncRNAs as well as opportunities and challenges they bring to clinics. **(A)** Functionalities of ncRNAs enable them with clinical potential and bring novel opportunities to clinics. Regulatory ncRNAs can function in chromatin remodeling, post-transcriptional regulation and transcriptional regulation. Based on these functionalities, experimental tools such as the CRISPR technique has been established to avail both research and clinics; besides, ncRNAs can capture dynamic subtle cellular changes pathological stimuli that are difficult to be precisely monitored using DNAs or proteins, and targeting miRNAs can effectively regulate many downstream target genes which is difficult to achieve using conventional strategies. **(B)** Several features of ncRNAs make clinical translation of ncRNAs challenging. NcRNAs are featured by promoting disease in one tissue but being protective in another, therefore, enabling tissue-specific drug delivery is of crucial importance when targeting ncRNAs in clinics. NcRNAs may be subjected to chronic loss-of-function adaptation, leading to inconsistencies observed between short-term inhibition and genetic deletion in some cases, and how to prevent drug efficacy decline with time imposes another big challenge. LncRNAs take advantages of both sequence matching and secondary and/or tertiary structures to take actions, and miRNAs can regulate multiple targets simultaneously, rendering it more important and complicated to prevent off-target effect. Oligonucleotide sequence may be toxic, which makes the safety issue more significant on drug delivery.

This review will critically assess the features and functionalities of ncRNAs with a focus on lncRNAs and miRNAs, identify their current applications and potential in cancer management including diagnostics, prognosis and therapeutics, proposes opportunities that ncRNAs may bring to complement the current diagnostic/therapeutic modalities for improved cancer control, and discuss challenges faced by bringing ncRNAs from academic frontier to the bedside in clinics as well as the potential solutions (Figure 1).

## Long Non-coding RNAs

### Features of lncRNAs

LncRNAs are, in general, >200 nts, and vary from 1,000 to 10,000 nts, some of which are upto 1,00,000 nts in length (14, 15). LncRNAs were firstly recognized in mice when people were attempting to sequence the full-length cDNAs (16). Ever since 2007 when *HOTAIR*, a 2.2 kilobase functional lncRNA, was identified to participate in several processes of epigenetic regulations (17), the mystery on the existence and prevalence of lncRNA that constitute genome redundancy was revealed, and a new regime of exploring the regulatory roles of lncRNAs was opened. With the development of transcriptomics, lots of lncRNAs were identified in the past decade as important functional products of the genome (15).

LncRNAs are highly diverse, with little shared feature regarding the localization, structure, mechanistics or mode of action across the entire mammalian lncRNA regime. However, lncRNAs are featured by low GC content, lack of introns and start codons (18). While some lncRNAs perfectly match Watson-Crick base-pairing, some employ imperfect pairing, and Watson-Crick and non-Watson-Crick base pairs are interspersed (14). These sequence traits enable them with some biological features such as nucleus positioning and low transcription activity (19). The secondary and tertiary structures of lncRNA play vital roles for them to take on any action modes such as protein recognition, catalysis and metabolite sensing (14, 20), where UV crosslinking and computational modeling have been used to characterize their functionalities in the 3D space (21–23).

Diverse approaches have been established to classify lncRNAs according to different features. LncRNAs can be categorized into 5 classes, i.e., sense lncRNA, antisense lncRNA, intronic lncRNA, intergenic lncRNA, and bidirectional lncRNA, based on their genomic locations and relative positions to protein-coding genes. Sense and antisense lncRNAs are, by their names, transcribed from the sense and antisense strand, respectively; while intronic lncRNAs are lncRNAs entirely transcribed from the introns of protein-coding genes, intergenic lncRNAs are transcribed within genomic intervals of neighboring protein-coding genes; lncRNAs are considered bidirectional if they were transcribed concomitantly with an adjacent protein-coding gene on the same strand (24). Based on the functionalities of lncRNAs exerted on DNA sequences, they can be categorized into cis- and trans-lncRNAs. While cis-lncRNAs regulate the expression of neighboring genes, trans-lncRNAs modulate those of the remote genes (25). Headway will be made toward more comprehensive understandings on the functionalities and mechanisms of lncRNAs with the advancement of high-throughput technologies that, ultimately, lead to more reasonable classifications of lncRNAs.

### Functionalities of lncRNAs

**Figure 2:**
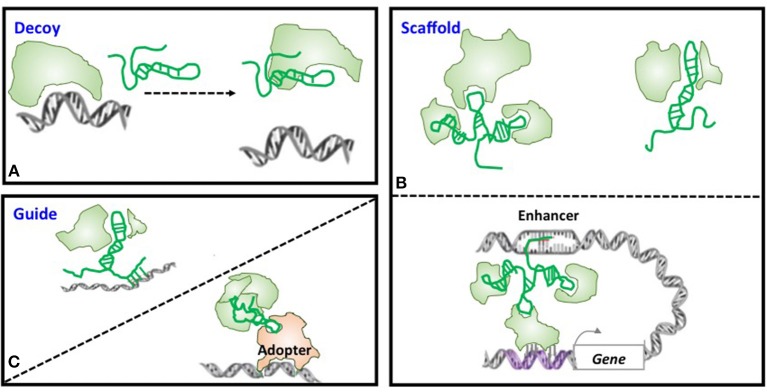
Models of lncRNA mechanisms of action. **(A)** Decoy. LncRNA can act as decoys that titrate away DNA-binding proteins such as transcription factors or regulatory RNAs such as miRNAs. **(B)** Scaffold. LncRNAs may act as scaffolds to bring two or more proteins into a complex or spatial proximity which, if through chromosome looping, can function as an enhancer. **(C)** Guide. LncRNAs may act as guides to recruit proteins such as chromatin modification enzymes to DNA.

Large number of lncRNAs have been recognized with the rapid advancement of experimental and computational technologies, with a small fraction of them being functionally annotated and even fewer been proved with *in vivo* functionalities. Through forming a RNA:DNA:DNA triplex, RNA:DNA hybrid (R-loop), RNA:RNA hybrid with a nascent transcript, or binding to a sequence-specific DNA binding protein, lncRNAs can function as decoys, scaffolds, and guides to interfere with and participate in various transcriptional and post-transcriptional programs (15, 26–28) (Figure 2). Functioning as decoys that block the access of other regulatory RNAs or proteins to the targeted DNA is the firstly identified mechanism of lncRNAs. For example, lncRNA *AK015322* functions as a decoy for miR-19b-3p to promote the proliferation of spermatogonial stem cells C18-4 (29); and lncRNA *Gas* releases the glucocorticoid receptor from DNA as a decoy to prevent transcription of metabolic genes under starvation conditions (30) (Table 1). LncRNAs can also serve as scaffolds to bring regulatory elements including proteins and RNAs together for collaborative functionalities including gene expression enhancement. For instance, lncRNA *GClnc1* rewires the histone modification pattern that ultimately contributes to gastric carcinogenesis through performing as a modular scaffold of the WDR5/KAT2A complex (58); the lncRNA *KHPS1* re-activates a poised enhancer of the proto-oncogene *SPHK1* via RNA-DNA-DNA triplex-dependent recruitment of epigenomic regulators E2F1 and p300 (59); and the lncRNA *Evf2* spatially organizes distant genes and functions as an enhancer in the developing forebrain (60) (Table 1). Last but not least is the “guide” role played by lncRNAs, with a classical example being *HOTAIR* that guides PRC2 in regulating the expression of a plethora of developmental and cancer-related genes (36), and another example being *lncRNA-21* that reroutes the nuclear factor hnRNP-K to specific promoters (61) (Table 1).

**Table 1:** Functionalities and mechanisms of example lncRNAs and miRNAs as biomarkers or therapeutic cancer targets.

**Name**	**Type**	**Functionality**	**Mechanism**	**References**
*AF075069*	lncRNA	Post-transcriptional regulation	mRNA turnover	(31)
*Bace1-AS*	lncRNA	Post-transcriptional regulation	mRNA turnover	(32)
*BC009800*	lncRNA	Post-transcriptional regulation	mRNA turnover	(31)
*BC058830*	lncRNA	Post-transcriptional regulation	mRNA turnover	(31)
*CCAT1*	lncRNA	Post-transcriptional regulation	miRNA sponge	(33)
*Falcor*	lncRNA	Transcriptional regulation	Transcribed within distal enhancer	(34)
*FER1L4*	lncRNA	Post-transcriptional regulation	miRNA sponge	(35)
*HOTAIR*	lncRNA	Transcriptional regulation	Interaction with histone methyltransferase	(36)
*H19*	lncRNA	Post-transcriptional regulation	miRNA reservoir	(37)
*H19*	lncRNA	Post-transcriptional regulation	miRNA sponge	(38)
*LincRNA-p21*	lncRNA	Post-transcriptional regulation	mRNA translation	(39)
*MALAT1*	lncRNA	Post-transcriptional regulation	mRNA splicing	(40)
*NEAT1*	lncRNA	Post-transcriptional regulation	miRNA sponge	(41)
*PTENpg1*	lncRNA	Transcriptional regulation	Interaction with DNA methyltransferase	(42)
*SPRY4-IT1*	lncRNA	Post-transcriptional regulation	miRNA sponge	(43)
*SRG1*	lncRNA	Transcriptional regulation	Transcribed within adjacent gene promoter	(44)
*Uchl1-AS*	lncRNA	Post-transcriptional regulation	mRNA translation	(45)
*XIST*	lncRNA	Transcriptional regulation	Interaction with histone methyltransferase	(46)
*XIST*	lncRNA	Post-transcriptional regulation	miRNA sponge	(47)
miR-193b	miRNA	Post-transcriptional regulation	Targeting gene	(48)
miR-200c-3p	miRNA	Post-transcriptional regulation	Targeting gene	(49)
miR-21	miRNA	Post-transcriptional regulation	Halt protein translation	(50)
miR-221/222	miRNA	Transcriptional regulation	Targeting lncRNA	(51)
miR-31	miRNA	Post-transcriptional regulation	Targeting gene	(52)
miR-372	miRNA	Post-transcriptional regulation	Targeting gene	(35)
miR-4286	miRNA	Post-transcriptional regulation	Targeting gene	(53)
miR-513b	miRNA	Post-transcriptional regulation	Targeting gene	(54)
miR-603	miRNA	Post-transcriptional regulation	Halt protein translation	(55)
miR-663a	miRNA	Transcriptional regulation	Targeting lncRNA	(56)
miR-92b	miRNA	Post-transcriptional regulation	Halt protein translation	(57)

#### Transcriptional Regulation

**Figure 3:**
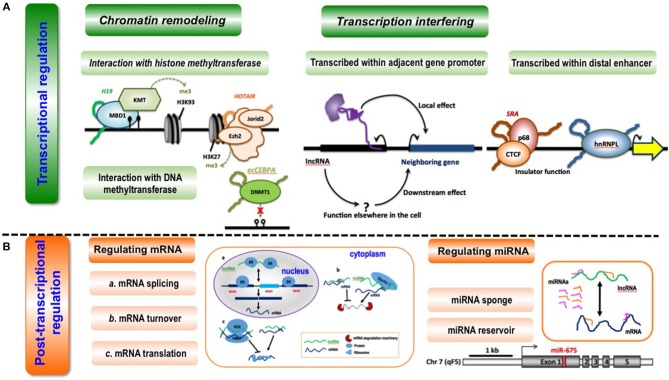
Conceptual scheme illustrating the primary functionalities of lncRNAs. **(A)** LncRNA-mediated transcriptional regulation can function through chromatin remodeling (via interaction with histone methyltransferase or DNA methyltransferase) and transcription interfering (via being transcribed within adjacent gene promoters or distal enhancers). Examples are given as below. During interactions with histone methyltransferases, lncRNA *H19* binds to the methyl-CpG-binding protein MBD1 to control gene expression by recruiting a histone lysine methyltransferase (KMT) (62); lncRNA *HOTAIR* interacts with the histone methyltransferase Ezh2, a key component of the PRC2 complex, to mediate chromatin-dependent gene regulation, and also interacts with Jarid2, a PRC2-associated factor, to promote the targeting of PRC2 to chromatin (63). During interactions with DNA methyltransferase, lncRNA *ecCEBPA* interacts with the DNA methyltransferase DNMT1 to block DNA methylation and control gene expression (64). When being transcribed within adjacent gene promoters, lncRNAs affect the expression of neighboring genes directly via local effect or indirectly via downstream effect (65). When being transcribed within distal enhancers, lncRNA *SRA* interacts with transcription factor CTCF and its associated DEAD-box RNA helicase p68 to form a complex that is essential for insulator function (66); lncRNA *THRIL* binds to hnRNPL, a component of hnRNP complexes, and the *THRIL*-hnRNPL complex regulates transcription by binding to target gene promoters (67). **(B)** LncRNAs can regulate post-transcription via regulating miRNAs or mRNAs. LncRNAs can modulate mRNA splicing, mRNA turnover and mRNA translation at the post-transcriptional level. LncRNA *MALAT1* competes for binding for splicing regulatory proteins SR to assist in pre-mRNA splicing (40); lncRNA *Bace1-AS* forms a hybrid with *Bace1* mRNA to prevent its decay (68), and lncRNAs such as *BC058830, AF075069, BC009800* promotes the decay of Alu-containing mRNAs (31); lincRNA-p21 interacts with partially complementary mRNAs of *Junb* and *Ctnnb* and suppress their translation via recruiting translation repressors Rck and Fmrp (39), and *Uchl1-AS* interacts with *Uchl1* mRNA via a SINEB2 sequence and a segment fully complementary with the 5′ end of the mRNA to recruit ribosomes and activate *Uchl1* mRNA translation (45). LncRNAs can function as a sponge or reservoir of mRNAs during post-transcriptional modulation. LncRNA *CCAT1* could function as a molecular sponge of let-7 and to reduce its suppression on the endogenous targets *Hmga2* and *c-Myc* in hepatocellular carcinoma (69); increased lncRNA *H19* is associated with decreased *Igf1R* mRNA expression, as miR-675 that targets *Igf1R* is embedded in the first exon of *H19* (37).

LncRNAs can regulate gene transcription via chromatin remodeling (Figure 3) through confining chromatin remodeling complexes to particular genomic regions via interactions between lncRNAs and histone methyltransferase PRC2 (polycomb repressive complex 2) (15, 26, 70). For example, the lncRNA *HOTAIR* induces H3K27me3 through direct interactions with EZH2 (the catalytic subunit of PRC2) that, ultimately, suppresses *HOXD* expression (71, 72). Another example is how lncRNA *Xist* regulates X chromosome dosage compensation in mammals, where *Xist* expressed from one X chromosome localizes PRC2 and H3K27me3 to the inactive X chromosome in female cells through physically interacting with PRC2 via RepA; and this results in altered chromatin structure of the entire inactive X chromosome and restored expression of genes located on this chromosome that were previously transcriptionally silenced (46). Besides, lncRNAs can utilize DNA methyltransferases to modify chromatin conformation (73–75). For instance, antisense lncRNAs such as *PTENpg1* was shown to interact with a 5′UTR-containing promoter-spanning transcript followed by recruitment of DNA methylatransferase 3A (DNMT3a) to epigenetically control the transcription of the *PTEN* pseudogene (42) (Table 1).

LncRNAs can regulate gene expression by interfering with the transcription of regulatory elements such as enhancers and promoters (76, 77) (Figure 3). Some lncRNAs are transcribed within adjacent gene promoters, and thus capable of modifying relevant gene expression through interfering the binding of transcription factors. For instance, the lncRNA *SRG1* is transcribed across *SER3* promoter, and *SER3* expression is considerably reduced on *SRG1* transcription (44) (Table 1). LncRNA can be transcribed within distal enhancers that can modulate the expression of neighboring genes through recruiting transcription factors to these loci (78, 79). Transcription factors are sensitive regulators of gene expression, deregulation of which at either transcriptional or translational levels may lead to life-threatening diseases including cancers. LncRNAs are spatially associated with TFs in the genome, and can regulate gene expression by interacting with TFs, with a recent example on the identified regulatory loop between FOXA2 (a transcription factor) and *Falcor* (a lncRNA) being reported. Specifically, the lncRNA *Falcor* represses the expression of TF FOXA2 through binding to its promoter that, in turn, regulates *Falcor* expression; and disruption of this *Falcor*-FOXA2 regulatory loop may lead to altered cell adhesion and migration that, ultimately, results in goblet cell metaplasia (34) (Table 1).

#### Post-transcriptional Regulation

LncRNAs can modulate multiple processes in the post-transcriptional modification of messenger RNAs (mRNAs), including pre-mRNA splicing, mRNA turnover and mRNA translation, through recognizing the complementary sequences (Figure 3). For instance, lncRNA *MALAT1* competes for the binding with splicing regulatory proteins SR to assist in pre-mRNA splicing (40); lncRNA *Bace1-AS* forms a hybrid with *Bace1* mRNA to prevent its decay (32), and lncRNAs such as *BC058830, AF075069, BC009800* promote the decay of Alu-containing mRNAs (31); lincRNA-p21 interacts with partially complementary mRNAs of *Junb* and *Ctnnb* and suppresses their translation via recruiting translation repressors Rck and Fmrp (39), and *Uchl1-AS* recruits ribosomes and activates *Uchl1* mRNA translation through short interspersed nuclear element B2 (SINEB2)-mediated interactions with *Uchl1* mRNA (45) (Table 1).

Some lncRNAs act as competing endogenous RNAs (ceRNAs) capable of sponging miRNAs to reduce their suppressive effects on targeted genes (80), which is common in tumorigenesis (Figure 3). For instance, the lncRNA *CCAT1* could elevate the expression of target genes *Hmga2* and *c-Myc* in hepatocellular carcinoma via sponging their negative regulator let-7 (33); the lncRNA *NEAT1* represses glioma progression and reduces its malignancy through sponging miR-107 and inhibiting *CDK14* (41); the lncRNA *SPRY4-IT1* promotes EMT of cervical cancer by sponging miR-101-3p (43); and the lncRNA *FER1L4* functions as a sponge of miR-372 that targets *E2F1* to regulate cell cycle progression in glioma cells (35) (Table 1). Also, lncRNAs can act as the reservoir of miRNAs that ultimately leads to gene repression via giving rise to miRNAs (Figure 3). For example, increased lncRNA *H19* is associated with decreased *Igf1R* mRNA expression, as miR-675 that targets *Igf1R* is embedded in the first exon of *H19* (37) (Table 1).

## Micro RNAs

### Features of miRNAs

MiRNAs are a group of petite ncRNA molecules ranging from 16 to 27 nt in length that are efficient to adjust gene expression transcriptionally and/or translationally. MiRNAs straightly interact with partial complementary target spots positioned in the 3′ untranslated region (UTR) of the targeted gene to repress its expression. Above 60% mRNAs have miRNA binding sites in their 3′UTR regions according to computational predictions, suggesting the critical roles of miRNAs in maintaining cellular homeostasis at both healthy and diseased states. Many miRNAs regulate up to hundreds of mRNAs, implicating complicated regulatory roles of miRNA on the topology of mRNA modulation network. The expression of miRNAs is tissue-specific (81), and tightly regulated temporally and spatially (82).

Generally, mammalian miRNAs are encoded in the genome and transcribed as initial miRNA transcripts (pri-miRNAs) through RNA Polymerase II, get processed to the precursor miRNAs (pre-miRNA) that harbor a stem-loop structure through the DROSHA-DGCR8 complex, and located to the cytosol through exportin5 (XPO-5); there, pre-miRNAs are processed further into dsRNAs that are nearly 21 nt in length by RNase III enzyme DICER1 coupled with PACT or TRBP in cytoplasm; short dsRNAs are merged into RNA-induced silencing complex (RISC) through binding with an Argonaute family member; while one strand of the dsRNA is preserved in RISC, the other stand undergoes fast degradation (83). The miRNA functions through binding to the 3′ UTR of the target messenger RNA (mRNA) via sequence complementarity (84). While perfect base pair match leads to mRNA degradation, imperfect pairing results in mRNA sequestration and translation inhibition (85).

MiRNAs undergo complicated post-transcriptional alterations such as miRNA accumulation, editing, processing and re-cycling inside P-bodies during maturation (86). This complex yet well-orchestrated miRNA maturation process renders it difficult to evaluate, in real time, the spatio-temporal pattern of miRNA expression and results in a gap between the expression and function of miRNAs. Therefore, understanding the functionalities of miRNAs under physiological and pathological conditions imposes a great challenge in the field of miRNA biology. Traditional miRNA profiling approaches such as microarray, real-time PCR, Northern blot, deep sequencing and *in situ* hybridization, though adding to our knowledge on miRNA biology, can rarely throw light on the spatiotemporal configurations and roles played by miRNAs *in situ*. Increasing attention has been paid to non-invasive molecular imaging methods which may potentially resolve these aforementioned issues (87).

### Functionalities of miRNAs

MiRNAs can regulate gene regulation at the post-transcriptional level via degrading targeted mRNAs or repressing protein translation, and at the transcriptional level via targeting regulatory RNAs such as lncRNAs.

#### Transcriptional Regulation

MiRNAs can target regulatory molecules such as lncRNAs that may affect gene expression via functioning as decoys, scaffolds, or guides; and quite often a regulatory axis involving miRNA, lncRNA and mRNA is implicated in the regulatory mechanism that drives the observed pathological consequence. For instance, miR-221/222 can inhibit tumor apoptosis in breast cancers via targeting the lncRNA *GAS5* (51) (Table 1). The miR-663a targets *ZBTB7A* that transcriptionally suppresses lncRNA *GAS5* under ER stress (56), and lncRNA *GAS5* suppresses tumor cell proliferation and EMT in oral squamous cell carcinoma via regulating the miR-21/Pten axis (88) (Table 1).

#### Post-transcriptional Regulation

Degrading target mRNAs at the post-transcriptional level is the common mechanism adopted by miRNA in gene regulation. The functional roles of miRNA in both physiological and pathological processes by adjusting their target gene expression have been demonstrated through vast number of evidences (89–91), with recent attentions attracted to the roles played by miRNAs in cancer initiation, progression, angiogenesis, metastasis and chemoresistance (92). For example, conditional knockout of miR-31 was shown to promote the development of colitis-associated cancers (52); miR-193b exhibited a tumor suppressive role in human esophageal squamous cell cancers via targeting *KRAS* (48); miR-200c-3p suppressed the proliferative and invasive abilities of nephroblastoma cells via targeting *FRS2* (49); miR-4286 and miR-513b each promoted cell proliferation and migration via targeting *Pten* and *HMGB3*, respectively, in non-small cell lung cancer (53, 54) (Table 1).

MiRNA can also inhibit protein translation. For instance, miR-603 promoted tumor proliferation by inhibiting the translation of BRCC2 protein in osteosarcoma (55); miR-21 repressed the translation of the tumor suppressor PDCD4 (50); and miR-92b restored the sensitivity of hepatocellular carcinoma to ionizing radiation-based radiotherapy through inhibiting the protein expression of p57kip2 (57) (Table 1).

## Clinical Implications of Non-coding RNAs

Variations in non-coding genomic regions have been associated with cancer susceptibility (93), and a plethora of cancer risk loci were documented to be transcribed into ncRNAs that play vital roles in tumorigenesis and progression.

### Diagnosis and Prognosis

LncRNAs can serve as diagnostic and prognostic biomarkers if they were highly correlated with particular cancer states. For example, the expression of lncRNA *PRNCR1* was elevated in the urine samples of prostate cancer patients, rendering it a fine non-invasive indicator of prostate cancers (94). Many other lncRNAs have been associated with various malignancies such as *MALAT1* in non-small-cell lung cancer and hepatoblastoma, *HOTAIR* in pancreatic and colorectal malignancies (95), *FOXD2-AS1* in colorectal cancer (96), *LSINCT5* in breast and ovarian cancers (97), *PTCSC3* in papillary thyroid cancer (98), *TUG1* in bladder urothelial cancer (99), and *UCA1* in squamous carcinoma (38, 100, 101), suggesting their diagnostic potential. However, we are still unclear on what alterations in the primary sequence of lncRNAs mean for their functionalities, and how to predict the activities of lncRNAs through their secondary structures and the properties of its interacting disease-associated proteins (102), answers to which may advance our understandings on the working mechanisms of lncRNAs that drive their prognostic roles.

Tumors ubiquitously exhibit dysregulated miRNA expression and such profiles convey useful information for tumor classification and prognosis. MiRNAs are present in numerous body fluids including saliva, plasma, serum, and amniotic fluid (103). Thus, extracellular miRNAs are potential biomarkers for disease diagnosis. Serum miRNAs have been associated with the incidence of both solid tumors and hematologic cancers, and proposed for early detection of various malignancies (104, 105). As miRNAs are featured by having multiple targets, they are typically used in panels for cancer diagnosis/therapeutics. A serum miRNA classifier consisting of 7 miRNAs (miR-29a, miR-29c, miR-133a, miR-143, miR-145, miR-192, miR-505) has been used for hepatocellular cancer early detection in a retrospective, longitudinal, multicenter biomarker identification study (106). The miRNA7™ panel was approved by CFDA for liver cancer diagnosis in August 2017, which becomes the first ncRNA panel translated into clinics and released on the market in the world. It outweighs the traditional alpha-fetoprotein (AFP) kit in being capable of identifying AFP-negative liver cancers from just 0.2 ml blood plasma with 84% sensitivity and 88% specificity. Another classifier consisting of 6 miRNAs, i.e., miR-20a-5p, miR-21-5p, miR-103a-3p, miR-106b-5p, miR-143-5p, and miR-215, was established to classify stage II colon carcinomas into patients having high and low risks regarding disease progression, and was demonstrated as a reliable prognostic tool for disease recurrence prediction (107). A microRNA-based signature (MiROvaR) comprised of 35 miRNAs was developed to predict disease progression or early relapse of epithelial ovarian cancers from a cohort study (108). Fourteen circulating miRNAs were identified associated with docetaxel chemotherapy response among prostate cancers from a phase I discovery study (that included 97 patients) (109), out of which six (miR-132, miR-200a, miR-200b, miR-200c, miR-375, miR-429) were verified with prognostic values from the following phase II validation study (that consisted of 89 patients) (110). Besides, circulating miRNAs have been correlated with diverse cardiovascular disorders (111). It is worth noting that the origin and functions of extracellular miRNAs, though being actively investigated, are not fully characterized. Whether circulating miRNAs in mammals exhibit hormone-like actions or achieve sufficient concentrations in certain tissues to repress distal targets remains unanswered. This, once addressed, can provide useful insights to avail clinical translation of miRNAs.

### Therapeutics

#### LncRNAs in Therapeutics

LncRNAs can be targeted to combat against disease progression and enrich current treatment modalities in the control cancer patient who developed resistant to traditional therapies. For example, lncRNA *aHIF* is an antisense RNA (aRNA) overexpressed in human renal cancer with drugging potential; *LINK-A* overexpression was observed in breast and lung cancer patients that had developed resistance to AKT inhibitors, providing the rational for targeting *LINK-A*, either alone or in combination with AKT inhibitors, in the treatment of such cancers (112).

A well-characterized function of lncRNAs is its modulatory roles on chromatin states (113). Thus, we can target aRNAs to achieve locus-specific up-regulation of a protein and its natural variants, e.g., by targeting *BDNF-AS*, the natural antisense transcript of *BDNF*, 2- to 7-folds up-regulation on *BDNF* expression with no visible effect on the neighboring genes was observed both *in vitro* and *in vivo* (114). To achieve this, single-stranded oligonucleotides, namely antagoNATs, can be fabricated to degrade these antisense transcripts and/or block their interactions with sense mRNAs (114) which, however, needs *in vivo* adaptations such as chemical modifications toward improved metabolic stabilities and minimized length for enhanced cellular uptake. The locked nucleic acids (LNA)-modified antisense oligonucleotides strategy was established accordingly. Brown et al. combined the carbamate DNA backbone analogs with the LNA (LNA-CBM) to confer enzymatic resistance and thermodynamic stability, and oligonucleotides modified with LNA-CBM exhibited increased stability in the presence of fetal bovine serum and snake venom (115).

LncRNAs can also be targeted, at least *in vitro*, using siRNAs if the targeted region in the antisense transcript did not directly overlap with the sense transcript. For example, through a loss-of-function RNA interference screen targeting 797 aRNAs, the functionalities of aRNAs against *CCPG1* and *RAPGEF3* in disrupting signalings orchestrating cell viability were revealed (116). It is worth mentioning that some aRNAs such as *BACE-AS* has high basal expression level and, can mask miRNA binding sites through forming duplexes with mRNAs; and siRNAs targeting these antisense transcripts will down- rather than up-regulate the targeted transcripts (117).

#### MiRNAs in Therapeutics

Dysregulation of miRNAs can be well-tolerated in normal tissues yet can profoundly alter cell behavior in response to pathologic stress. Thus, miRNA offers a potential tool to perturb a disease progress without creating unexpected adverse effects in normal cells. The antagoNAT that is modified with LNA through a covalent bridge connecting the 2'-oxygen and 4' carbon of the ribose moiety of the nucleotide can form strong duplexes with its target miRNAs, sequester these targeted miRNAs but do not promote their degradation. Tiny LNAs were later created to inhibit miRNA families sharing homology by targeting their seed regions (118). AntagoNATs modified with LNA can be systemically delivered by injection with little or no toxicity, with sufficient uptake and desirable therapeutic efficacy being reported in, e.g., immune, vascular and heart systems in rodents. An LNA-modified antagoNAT against miR-122 (namely SPC3649) was developed to fight against hepatitis C virus infection, where a dose-dependent prolonged viral reduction was observed after 4 weeks of treatment without observable toxicity from a human Phase II study (119). TargomiRs are minicells carrying miR-16-based mimic miRNAs targeting EGFR, which counteract recessed expression of miR-15 and miR-16 family miRNAs in malignant pleural mesothelioma by design; and a phase I, open-label, dose-escalation study assessing the safety and activity of TargomiRs was conducted in patients carrying malignant pleural mesothelioma, with positive results being reported (120) (Table 2, Supplementary Table 1).

**Table 2:** Example clinical trials involving miRNAs as cancer biomarkers.

**Name**	**Panel size**	**Study type**	**Study size**	**Clinical use**	**Cancer type**	**References**
7-miR panel	7	Multicenter, retrospective, longitudinal study	257 patients for discovery, 352 and 139 patient from two cohorts for validation	Early detection	Hepatocellular carcinoma	(106)
6-miR panel	6	MicroRNA expression analysis	40 patients for discovery, 138 for validation	Risk of disease progression	Stage II colon cancer	(107)
MiROvaR	35	Cohort study	179 patients for discovery, 263 and 452 patients from two cohorts for validation	Early relapse or progression	Epithelial ovarian cancer	(108)
6-miR panel	6	Phase II clinical trial	2 cell lines for discovery, 97 patients for validation	Early relapse or progression	Prostate cancer	(110)

As many miRNAs appear to be beneficial rather than pathologic, the development of miRNA mimics represents another important therapeutic regime. Through encapsulating or conjugating synthetic miRNA duplexes inside/to nanoparticles such as liposome or using adeno-associated virus as the delivery vehicle, successes (recessed tumor growth without obvious toxicity to normal cells) in miRNA mimicry-based therapeutics have been achieved in many tumor animal models (121, 122). However, the use of injectable, naked miRNA mimics, unlike miRNA inhibitors, remains problematic, as the complementary strand needs to be modified to facilitate stability and cellular uptake but has the potential to act as an anti-miRNA.

## Opportunities Brought by ncRNAs to Cancer Control

The regulatory roles of RNAs, in particular ncRNAs, on gene expression has led to the establishment of experimental tools such as RNA interference and CRISPR/Cas9 for gene modulation. The functions of ncRNAs in transducing signals of multiple core cancer pathways such as Hedgehog, JAK/STAT, WNT, HIF, NFkB, PI3K/PTEN, result in their translational applications in both diagnosis and therapeutics. As prognostic biomarkers, ncRNAs may capture dynamic subtle alterations due to pathological stimuli that are difficult to be precisely monitored using DNAs or proteins. Therapeutically, targeting ncRNAs offers us a direct tool to inhibit protein function or down-regulate gene expression and represents a promising strategy to up-regulate endogenous genes in a locus-specific manner. That is, inhibiting miRNAs using antagoNATs can effectively up-regulate many downstream target genes, which is difficult to achieve using conventional strategies. This is therapeutically advantageous as it is easier to inhibit rather than up-regulate a drug target where the latter typically requires the identification of appropriate agents; and conventional strategies that restore reduced gene expression or recessed protein functionalities possess various limitations including, e.g., a need of lifespan intake of therapeutic proteins that may be associated with adverse immune response by themselves, and difficulty in producing synthetic proteins that can fully mimic the plethora of endogenous counterparts of targeted proteins.

## Challenges Faced by ncRNAs in Cancer Control

Many challenges must be overcome to fully convey the promise of ncRNA-based therapeutics. First, targeted and efficient delivery of oligonucleotide-based therapies, particularly anti-miRNA, miRNA mimics, siRNA, and shRNA, into tissues and cells remains a crucial hurdle. This is worthy of special attention as ncRNAs especially miRNAs may accelerate disease in one tissue and be tumor suppressive in another, e.g., miR-26a promotes gliomagenesis (123) but shows double-sided roles in the carcinogenesis of hepatocellular cancers (124, 125). Second, safety imposes an important challenge before drugging ncRNAs can be clinically applicable. Oligonucleotide sequence has been reported to have adverse effects due to, mostly, protein binding. For instance, phosphorothioate-containing oligonucleotides show pro-inflammatory properties. More preclinical and clinical studies are needed to decipher such correlations between sequence motifs and such liabilities. Off-target is another source of unanticipated events, which warrants our consideration from multiple aspects in the design of oligonucleotide-based drugs including, e.g., RNA structure, its association with various proteins, mismatch in the cleavage site etc. Third, the effects of ncRNAs may vary among cancer types, e.g., lncRNA *Xist* is tumor suppressive in osteosarcoma (126), cervical cancer (8), and breast tumor (127), but is oncogenic in pancreatic cancer (47), gastric cancer (128), thyroid cancer (129), colon cancer (130), hepatocellular carcinoma (131), non-small-cell lung cancer (132), bladder cancer (133), glioblastoma (134), and nasopharyngeal carcinoma (135). Fourth, there exists an inconsistency between short-term chemical inhibition and genetic deletion, complicating our understanding toward the functionalities of ncRNAs in relation to disease control. For instance, despite the pathological associations they demonstrate, germ-line depletion of miR-21 does not affect cardiovascular pathology, *H19*-knockout mice does not show a disease phenotype (136), *HOTAIR*-deletion does not abolish *PRC2* targeting (137), and *MALAT1* and *NEAT1* removal yields normal and viable mice (138, 139). This indicates that chronic loss-of-function of ncRNA may lead to adaptive responses to compensate for its absence, which makes our last concern, i.e., whether therapeutic efficacy of drugs targeting ncRNAs will decline with time due to the compensation of the target network for diminished ncRNA activity, challenging.

It is also important to address the differences between targeting lncRNAs and miRNAs. First, aRNAs, a type of lncRNAs, are primarily chromatin modulators suppressing transcription in the nucleus, whereas miRNAs are post-transcriptional repressors affecting mRNA stability in the cytoplasm. Second, antagoNATs can be used to target aRNA through cleaving or blocking a RNA or protein target, whereas steric blockade is typically used to inhibit miRNA. Third, there are much more yet less-studied lncRNA species than miRNAs. Fourth, cis-acting aRNAs are gene-locus specific, whereas miRNAs can affect the stability of many mRNAs. As several problems faced by drugging miRNAs naturally vanish when targeting lncRNAs, lncRNAs deliver more clinical promises than miRNAs despite the fact that they are less-well studied for clinic translation. For example, it is difficult to establish a precise correlation between miRNA target engagement and therapeutic efficacy as miRNAs can moderately modulate myriad targets to evoke their effects and oligonucleotides may be sequestered in various cellular compartments to create further uncertainties; such a problem does not exist in the case of lncRNAs given their working mechanisms.

## Perspectives

LncRNAs and miRNAs each represent a big class of ncRNAs with profound clinical implications, but differ greatly in terms of the nuclear acid biology, cellular behaviors and working mechanisms. Though both functioning through perfect or imperfect base-pairing with DNAs, RNAs and proteins, lncRNAs can take on more diversified action modes such as decoy, scaffold and guide which are all derived from their long length and the tertiary structure they can form. While lncRNAs can regulate gene expression in both directions, miRNAs, on the other hand, represent a class of sole negative gene expression regulators either through degrading target mRNAs or halting the translation of target genes; however, the regulatory flexibility of miRNAs can be largely extended by targeting lncRNAs, forming regulatory miRNA-lncRNA axes orchestrating the spatial and temporal cellular behavior under both normal and pathological conditions (140–145).

The identified mechanisms are not mutually exclusive and one ncRNA may have multiple working mechanisms under different circumstances. For example, the lncRNA *H19* can function as a miRNA reservoir of miR-675 that suppresses cell growth (37) and as a miRNA sponge of let-7a that up-regulates cyclin D1 expression (146); the lncRNA *XIST* interacts with histone methyltransferase at the transcriptional level in regulating chromosome dosage compensation (46), and sponges miR-34a to promote colon cancer progression at the post-translational level (130).

Other types of ncRNAs may also function as disease indicators or therapeutic targets. For instance, deficiency in SNORD50A/B snoRNAs can augment the functionalities of K-Ras that ultimately leads to hyperactivated MAPK/ERK signaling and carcinogenesis; and SNORD50A/B deletion occurs at a frequency of >10% in each of 12 common cancers. Therefore, exploring the varieties of ncRNAs, and functionalities and clinical use of novel types of ncRNAs represent another promising research domain for improved cancer management.

NcRNAs have offered a plethora of opportunities toward delicate gene modulations and precision medicine. Challenges faced by bringing ncRNAs to the bed-side could be well-resolved only if multi-domain knowledges and techniques were intelligently brought together. Nanomaterials such as gold nanoparticles (AuNPs), carbon-based nano-composites, and liposome could help to achieve targeted and efficient delivery of ncRNAs given their flexibility in surface modification, size and hydrophobicity control, as well as other physiochemical features that can, e.g., protect cargoes from fast degradation. For instance, by encapsulating a miRNA antagoNAT against miRNA-122 harboring 2′-OMe and phosphorothioate modifications (namely, AMO122) inside YSK05-MEND that is a multifunctional nano-vehicle (MEND) containing a pH-sensitive lipid YSK05, more liver deposition and drug efficacy were observed for YSK05 carried by MEND than free YSK05 that ultimately led to enhanced liver cancer treatment efficacy *in vivo* (147). Computational approaches and high-throughput technologies could be utilized to resolve issues related to seemingly unpredictable effects of ncRNAs in different tissues by drawing a comprehensive picture on the spatio-temporal regulatory properties of a given ncRNA.

As being consistent with many biological networks, the experimentally derived RNA interactome is scale-free (148). A scale-free network is biologically plausible given its robustness to extra- or intra- cellular perturbations and evolutionary economy in pertaining functionalities. In such networks, the number of nodes is negatively correlated with that of interactions involved, formulating a hierarchical structure. A failure is more likely to hit a node with fewer degrees rather than a hub. In addition, a scale-free network is organized such that low-degree nodes are condensed in sub-networks that are connected by a few promiscuous nodes; thus, a given functionality can be easily reached by convolving small number of players through multiple paths. On the contrary, promiscuous nodes are prevalent in a flat network, and have been reported in many pathological events such as gene fusions (149, 150). Thus, a scale-free network may represent an ordered and homostatic state, and a flat network may be indicative of a chaotic and diseased state. Toward this end, alterations in a network's structure may suggest a switch between ergodic sets and the associated cell states, and we may consider selecting nodes or connections capable of toggling a network's structure as candidates for subsequent clinical translations.

## Conclusions

With the rapid development of various bioinformatics technologies, a plethora of ncRNAs and their roles in tumorigenesis have been uncovered. Abnormally expressed ncRNAs might be adopted as diagnostic/prognostic biomarkers, and therapeutic targets for cancer management. More studies investigating novel mechanisms of ncRNAs in mediating cancer initiation and progression are needed for comprehensive understandings on their roles in carcinogenesis, and solutions to challenges faced by translating ncRNAs into clinics are imperatively needed to embrace the new paradigm shift from protein coding to non-coding RNA regime. Toward these goals, integrative efforts from multiple domains such as nanomaterials, computational system biology, molecular biology, and clinical medicine should be intelligently brought together.

## Author Contributions

XD conceptualized the ideas and prepared the figures and tables. XD and AK conducted the literature search and drafted the manuscript. XD and JZ offered financial support. All authors have read and approved the manuscript.

### Conflict of Interest

The authors declare that the research was conducted in the absence of any commercial or financial relationships that could be construed as a potential conflict of interest. The handling editor declared a past co-authorship with one of the authors XD.
