# Supplementary material for: The Emerging Role of Major Regulatory RNAs in Cancer Control
Source: Front Oncol. 2019 Sep 24;9:920. doi: 10.3389/fonc.2019.00920 (PMC6771296; doi:10.3389/fonc.2019.00920)
Supplement: Supplementary file 1 [file Table_1.pdf]

Supplementary Table 1. List of example clinical trials involving miRNAs as cancer biomarkers.

miRNA	MiROvaR	7-miR panel	6-miR panel 1	6-miR panel 2
miR-100-3p	Yes			
miR-103a-3p			Yes	
miR-106b-5p			Yes	
miR-132				Yes
miR-133a		Yes		
miR-135b-5p	Yes			
miR-141-3p	Yes			
miR-143		Yes		
miR-143-5p	Yes		Yes	
miR-145		Yes		
miR-151a-3p	Yes			
miR-192		Yes		
miR-193a-5p	Yes			
miR-193b-5p	Yes			
miR-195-3p	Yes			
miR-200a				Yes
miR-200a-3p	Yes			
miR-200b				Yes
miR-200b-3p	Yes			
miR-200c				Yes
miR-200c-3p	Yes			
miR-20a-5p			Yes	
miR-215			Yes	
miR-21-5p			Yes	
miR-23a-5p	Yes			
miR-29a		Yes		
miR-29a-5p	Yes			
miR-29c		Yes		
miR-29c-5p	Yes			
miR-30b-3p	Yes			
miR-30d-5p	Yes			
miR-330-3p	Yes			
miR-375				Yes
miR-423-5p	Yes			
miR-429	Yes			Yes
miR-452-5p	Yes			
miR-484	Yes			
miR-486-5p	Yes			
miR-505		Yes		
miR-506-3p	Yes			
miR-507	Yes			
miR-508-3p	Yes			
miR-509-3p	Yes			
miR-509-5p	Yes			
miR-513a-5p	Yes			
miR-513b-5p	Yes			
miR-514a-3p	Yes			
miR-574-5p	Yes			
miR-592	Yes			
miR-769-5p	Yes			
miR-890	Yes			
miR-99b-5p	Yes			
